# Barriers and facilitators for implementation of a complex health services intervention in long-term care homes: a qualitative study using focus groups

**DOI:** 10.1186/s12877-021-02579-y

**Published:** 2021-11-04

**Authors:** Rieka von der Warth, Vanessa Kaiser, Christina Reese, Boris A. Brühmann, Erik Farin-Glattacker

**Affiliations:** Present Address: Section of Health Care Research and Rehabilitation Research, Medical Center – University of Freiburg, Faculty of Medicine, University of Freiburg, Hugstetter Str. 49, D-79106 Freiburg, Germany

**Keywords:** Barriers, Facilitators, Nursing homes, Focus groups, Qualitative study, Coordinated medical care, Elderly, Primary care, Complex health intervention, Implementation research

## Abstract

**Background:**

With rising numbers of elderly people living in nursing homes in Germany, the need for on-site primary care is increasing. A lack of primary care in nursing homes can lead to unnecessary hospitalization, higher mortality, and morbidity in the elderly. The project CoCare (“coordinated medical care”) has therefore implemented a complex health intervention in nursing homes, using inter alia, regular medical rounds, a shared patient medical record and medication checks, with the aim of improving the coordination of medical care. This study reports upon the results of a qualitative study assessing the perceived barriers and facilitators of the implementation of CoCare by stakeholders.

**Methods:**

Focus group interviews were held between October 2018 and November 2019 with nurses, general practitioners and GP’s assistants working or consulting in a participating nursing home. A semi-structured modular guideline was used to ask participants for their opinion on different aspects of CoCare and which barriers and facilitators they perceived. Focus groups were analyzed using qualitative content analysis.

**Results:**

In total, *N* = 11 focus group interviews with *N* = 74 participants were conducted. We found six themes describing barriers and facilitators in respect of the implementation of CoCare: understaffing, bureaucracy, complexity, structural barriers, financial compensation, communication and collaboration. Furthermore, participants described the incorporation of the intervention into standard care.

**Conclusion:**

Barriers perceived by stakeholders are well known in the literature (e.g. understaffing and complexity). However, CoCare provides a good structure to overcome barriers and some barriers will dissolve after implementation into routine care (e.g. bureaucracy). In contrast, especially communication and collaboration were perceived as facilitators in CoCare, with the project being received as a team building intervention itself.

**Trial registration:**

WHO UTN: U1111–1196-6611; DRKS-ID: DRKS00012703 (Date of Registration in DRKS: 2017 Aug 23).

**Supplementary Information:**

The online version contains supplementary material available at 10.1186/s12877-021-02579-y.

## Background

Demographic transition is in full swing in Germany, with every fifth person being over 66 years old [1]. About 2 million people in Germany are in need of long-term care [2] and approximately 700.000 people were already living in NHs in Germany in 2018 [2].

Overall, older age was shown to be associated with greater health care utilization compared to the general population [3, 4]. Yet, the level of care needed was negatively associated with the probability of utilizing medical specialists, showing inequality in the health care system [5]. Other reports show a lack of primary care in NHs [6], leading to unnecessary hospitalizations for the elderly [7–9]. Unnecessary hospital admissions were not only shown to be costly [8, 10], but also increased the risk of complications and mortality in NH residents [11, 12]. The insufficient availability of general practitioners (GPs) and acute care were identified to be the main reasons for unnecessary hospital admissions in several studies [8, 13].

**Fig. 1:**
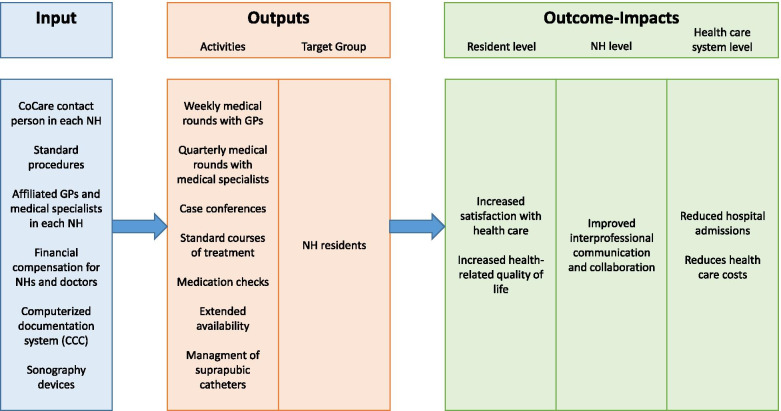
Logic Model of all modules of the CoCare intervention and the expected outcomes; GP = General practitioner; NH = nursing home

The project CoCare (“coordinated medical care”) aims to bridge the gap by implementing a complex health care intervention to improve primary care in NHs [14]. The study takes place in the federal state of Baden-Wuerttemberg, Germany, with 31 NHs implementing the new intervention, providing it to approximately 1150 residents. Furthermore, 25 NHs providing survey data will be used as control group and additionally claims data of *N* = 8000 of NH residents in Baden-Wurttemberg will be randomly drawn upon for comparison. Inter alia, the intervention provides a shared patient medical record entitled CoCare Cockpit (CCC) and standard courses of treatment, as operating procedures for nurses and GPs. Furthermore, CoCare financially compensates weekly on-site medical rounds, regular medication checks, case conferences and the extended availability of GPs. CoCare also aims at improving communication and collaboration between GPs and nurses by forming teams of physicians sharing care duties and by appointing a CoCare contact person in every NH. See Fig. 1 for an overview of the project.

CoCare was implemented by kick-off events in each NH with nurses and GPs attending so that they could be given information about the content and the process of the intervention. Furthermore, training regarding the use of the CCC, the standard courses of treatments and the management of suprapubic catheters were offered. As a result of CoCare, an increase in quality of care and care efficiency due to the intensified health care are expected. Furthermore, due to less unnecessary hospital admissions of NH residents, a reduction in health care costs is hypothesized. The intervention was delivered between January 2018 and September 2020, including NHs in both rural and urban areas to achieve better representativeness. Further information on the project and its study design has been published elsewhere [14].

In accordance with the guidelines for complex interventions by Moore et al. [15], a formative process evaluation has been conducted for this project. Acquiring specific information on the implementation process is crucial for decision makers before implementing the intervention on a larger scale. A process evaluation will therefore investigate how the fidelity, dose, adaption and reach of a newly implemented complex health intervention are and will further investigate the underlying theory of change [16, 17]. In CoCare several approaches were chosen to conduct the process evaluation, such as a) quarterly telephone interviews, b) annual audits with the nursing management to assess the fidelity and c) focus group interviews with NH residents to assess the reach of the intervention. Furthermore, focus group interviews with GPs, nurses and GP’s assistants were conducted to assess barriers and facilitators for implementation. The team conducting this process evaluation is part of a bigger CoCare project team, with the Association of Statutory Health Physicians Baden Wuerttemberg (KVBW) providing the CoCare infrastructure, and the Centre for Geriatric Medicine and Gerontology, Faculty of Medicine and Medical Center – University of Freiburg providing expertise in long-term care. Regular project meetings of all these participants were conducted to monitor the implementation process. At the end of the project, a summative evaluation will be conducted, based on claims data and questionnaires, addressing the costs of care, the quality of life of residents and satisfaction with the medical care provided.

This paper seeks to describe the results of the focus group interviews with GPs, nurses and GP’s assistants describing barriers and facilitators of the implementation process.

## Methods

This research was funded by the Innovation Committee at the Federal Joint Committee (G-BA), Germany under grant number NVF1_2016–080. The process evaluation was conducted at the Medical Center – University of Freiburg. Ethical approval for this study was granted by the Ethics Committee of the University of Freiburg (Approval Number: 333/17). The study was registered at the German Clinical Trial Register (DRKS00012703). Results are reported complying with the Consolidated criteria for reporting qualitative research (COREQ) checklist [18].

### Study design

Focus group interviews were conducted in mixed groups with GPs, GP’s assistants consulting a NH and nurses working in a NH enrolled in CoCare. Participants in each focus group were from the same NH and knew each other. Participating NHs were asked to send nurses, GPs and GP assistants engaged in CoCare. Focus groups were conducted between October 2018 and November 2019. All participating NHs were asked to participate in the focus groups. However, most NHs could not guarantee a sufficient sample size per focus group (5–6 participants) and thus, focus groups were only conducted in *N* = 11 NHs. Participation was voluntary and no disadvantages arose from non-participation. Participants were supplied with information on the objectives of the focus group interviews and written informed consent was obtained from all participants. The planning of this qualitative study, analysis of data and the interpretation of the results was solely done by the authors of this manuscript. As the authors used various methods for the evaluation of CoCare [14], some of the participants and researchers knew each other from previous contact.

Focus groups were conducted using semi-structured modular guidelines, which were chosen as CoCare comprises many modules. Using a modular guideline, participants were able to pick which of the many aspects from the intervention they felt were particularly important to discuss. The interview guidelines were developed for this study by the authors based on the study design of CoCare [14]. For this purpose, the CoCare modules served as main themes for the interview guidelines, asking questions about the benefits, barriers and facilitators for implementation in each module (e.g. Medical Rounds*: What benefits did you experience with the CoCare-rounds?).* The development was done in an iterative process, with the project team discussing and changing the interview guidelines until all team members agreed on it. Each interview began with a short introduction by the researcher, followed by a set of predetermined discussion topics (CoCare Modules) to choose from, based on the participants’ experience with the modules. Participants were asked to agree on topics they wanted to talk about, based on their experience of barriers/facilitators. The researcher then asked open-ended questions about the chosen topics. The interview guidelines had been previously published in Brühmann et al. [14] and can further be seen in Additional File 1.

Focus groups were conducted by CR and BB within the NHs. All of the focus groups were digitally recorded and transcribed verbatim by an external service provider. The transcripts were pseudonymized and given a consecutive ID-number from 1 to 11. As the focus groups were held in German, participants’ quotes to illustrate the finding were translated into English by the authors of this manuscript.

### Data analysis

Data analysis was based on the content analysis outlined by Mayring [19] using a mix of deductive and inductive approaches. For this purpose, an initial coding system was developed based on the CoCare modules from the modular guideline to deductively identify results according to the concept of the intervention. VK and RvdW then analyzed the first three interviews independently, using the initial coding system and taking notes as necessary. VK and RvdW adapted the coding system to the findings to account for the perceived importance of themes by participants, and discussing the results. This process was repeated twice before generating the final coding system. RvdW then coded the remaining focus groups using the final coding system. The results were presented to and discussed with the project team at different stages of the data analysis to ensure intersubjective comprehensibility. In this report, only barriers and facilitators for implementation described by participants will be displayed and discussed. As the focus groups focused on the team experience of CoCare, differences between professions were not analyzed. Data management was done using MAXQDA 2020 [20].

### Researcher characteristics

RvdW is a female researcher in the field of health services research and rehabilitation research. She holds a degree in psychology and has experience with qualitative studies. VK is a female master’s student in psychology and works as a student assistant in the field of health services research. Both, CR and BB are postdoctoral researchers in the field of health services research and rehabilitation research. CR is a trained female psychologist, and BB (male) holds two degrees in health sciences. EF is a male full time professor in health services research and rehabilitation research. His work focuses on methods in health services research.

## Results

### Overview of focus groups and participants

*N* = 11 focus groups with a total of *N* = 74 participants were conducted. Seven focus group interviews were conducted in non-profit NHs and four in private administrated NHs. The focus group sessions lasted between 20 and 59 min, with an average of 39 min. 78.8% of all participants were female. Most of the participants were nurses working within the participating NHs (*n* = 48), followed by consulting GPs (*n* = 20). A full overview of the distribution of participants can be seen in Table 1.

**Table 1:** Overview of participants

	Male	Female	***Total***
**General practitioners (GPs)**	13	7	*20*
**GP assistants**	0	6	*6*
**Nurses at the nursing homes**	3	45	*48*
***Total***	*16*	*58*	*74*

### Barriers and facilitators

In total, we found four themes describing barriers for the implementation of CoCare: “understaffing”, “bureaucracy”, “complexity”, and “structural barriers”. In some cases, participants described how they overcame perceived barriers. As facilitators, participants perceived “communication and collaboration” and “financial compensation”. Furthermore, the need of incorporation into standard care was described concerning the implementation of CoCare.

### Understaffing

According to the participants, one of the biggest barrier for frictionless implementation of CoCare was the lack of available human resources to devote to the intervention. In accordance, the issue of understaffing within the nursing team due to sickness or training was repeatedly mentioned.

*„It would be possible during normal operation. But then? Someone gets ill, someone is absent, someone is in training. And then suddenly nobody is here or nobody, who can….nobody has time to do these additional tasks.” (Focus group 1).*

Furthermore, understaffing was a barrier for the extended availability of GPs during the evening. Whilst extended availability even in the evening was natural for some GPs and NHs, some GPs would not do it due to the high workload. All participants agreed that, in order to ensure extended accessibility, more GPs supplying medical care to a NH would be needed to distribute the workload better.

### Bureaucracy

Participants mentioned that there was a great deal of bureaucracy in CoCare itself, which accounted for the high workload. It was not acceptable on a day-to-day basis for nursing staff to have to fill out various forms with residents before they were included in the study. In addition, one nursing home mentioned that it was a disproportionate amount of work to sort through documents for the project and do other formal things for CoCare. Thus, some participants feared that if CoCare will be implemented in standard care, it would pose a bureaucratic hurdle.

Bureaucracy was also mentioned concerning the CCC as NHs and GPs already use their own documentation system in order to comply with regulatory standards. Thus, NHs used up to three different systems including the CCC resulting in double documentation. This double documentation could potentially lead to mistakes, especially as no software interfaces are available.

*„And if you ask me, that would lead to mistakes, because every normal person, when they have written or typed the exact same thing for the third time, they might leave something out, no longer want to…(several other participants agree in the background)…every normal person would do that…” (Focus group 11).*

The double documentation resulted in most NHs only doing basic documentation in the CCC, with the medical rounds and medication being the most documented modules of CoCare.

### Complexity

With CoCare being a complex health intervention, the complexity of it as barrier was discussed in respect of several modules of CoCare. For instance, the standard courses of treatment were too detailed to learn and follow. Moreover, some participants did not know that the standard courses of treatment were available as a hard copy outside the CCC. Furthermore, case conferences as well as quarterly and annual meetings had not yet been implemented in most NHs, as participants struggled to distinguish between the three kinds of meetings. One participant mentioned that they had been conducting quarterly talks within the framework of the regular medical rounds.

*„So the medical rounds usually take a long time and the quarterly talk is supposed to be 90 min I think. I think we do that several times per quarter during the medical rounds, right?” (Focus group 4).*

However, it was also described that the complexity of the intervention could be overcome by combining two modules. For instance, participants stressed the importance of the medication checks and therefore combined them with the regular medical rounds in discussion with attending nurses. Where possible, GPs included psychiatrists and neurologists to talk about psychotropic drugs, too.

*“But now, sitting down during the visits, looking at the medication plan together and talking about it with every profession. […]” (Focus group 9).*

### Structural barriers

Participants reported some structural barriers for the implementation of CoCare. For instance, NHs struggled with their access to the internet and new network cables had to be installed before the CCC could be used. For some NHs this took a long time, especially when renovation was necessary.

Furthermore, all participants were of the opinion that medical specialists’ consultations, such as psychiatrists or urologists, are important. However, most participants said that it was not easy to find specialists for regular consultations within the NH. Only one NH reported that they had sufficient collaborations with specialists.

*“No other NH believes us when I tell them that we have a neurologist who comes to see our residents regularly. That’s not something you see very often.” (Focus group 1).*

Additionally, the affiliated GPs had to specifically order a sonography device if they wished to perform the management of suprapubic catheters and some participants mentioned that the delivery took too long. Furthermore, participants criticized that the sonography device can only be used on patients, who consented to take part in CoCare and not for other procedures.

*„In CoCare they only play a role for these catheters…[…]. Apart from that we don’t use it really. However, we could use it for other procedures.” (Focus group 9).*

### Communication and collaboration

Communication and collaboration was perceived as both, facilitator for the implementation of CoCare and a result of the project. This means, many participants saw CoCare as a team building intervention, improving the treatment of residents. Due to the strict framework of the project and its standard procedures, room for interprofessional communication is given.

For nurses, the access to GPs improved, making it easier to agree on a resident’s treatment. Participants said teamwork had become more professional in some NHs now, resulting in less conflicts and that decisions were now made with the whole team being involved. Participants also regarded a consistent contact person, mainly embodied by the care management of the NH, as positive.

*„When I come here, I have a number I can dial, I have someone who has time for me and is my contact person. Furthermore, I need to say: There is some structure. When something is not clear, there is someone I can talk to.” (Focus group 2).*

Moreover, the contact to the GP’s assistants was perceived as better and more structured, making it easier to organize treatment and medical rounds.

One participant mentioned that the improved communication and structured arrangements within the team might lead to the prevention of unnecessary hospitalizations.

*“There is always someone available and we have also agreed that we will be called first, before the emergency doctor or the ambulance, and we will then come and see whether it is really necessary.” (Focus group 1).*

Furthermore, GPs appreciated the newly set up GP teams for providing consultation at a NH, as the planning of visits and treatment were made easier. When GPs worked in a team at a NH, they left a message for the GP to lead the next visits or short consultations about the treatment planning. In this way, a continuous treatment of residents was guaranteed. However, GPs would not change the medication plan for a patient if the patient was being mainly treated by another GP. The newly built GP teams were also a relief for the nurses, as they knew a GP from the team would be at the NH to provide care more often than before.

### Financial compensation

A positive comment was made about the additional financial compensation CoCare provided to GPs for supplying medical care in NHs. This has made it easier to recruit GPs for the project.

*“…and now with the project there is the financial compensation, that needs to be mentioned. It gives us the opportunity to extend the care, which we do, and not do things on a minimal level.” (Focus group 7).*

Even though NHs equally received a financial compensation for their participation in CoCare, it was mentioned neither as a barrier nor as a facilitator for the project implementation.

### Incorporation into standard routine

CoCare is a complex health intervention with many modules. Thus, participants in all focus groups mentioned that the implementation process and incorporation into standard care took some time. It was therefore not possible to implement all modules at once. As the NHs entered the project at different times, the state of implementation differed across facilities. However, even if single components had not yet been implemented in the NHs, the participants did emphasize that CoCare builds awareness for the health care of the elderly and specific topics, such as the medication checks.

*Participant 1: “And there are a few things we still need to try. That’s too early to discuss.”*

*Participant 2: “So, especially the documentation, the CCC, that’s something new. We still kind of tiptoe around it. It is still very new and we need to gain some experience with it during the coming weeks and months.” (Focus group 4).*

However, some mentioned that modules, such as the medical round, were part of their regular routine even before CoCare. Where medical rounds had to be implemented, the regular scheduling facilitated the implementation and incorporation of CoCare into standard care. Participants mentioned that holding rounds on the same day each week allowed for better preparation by both, nurses and GPs. According to some participants, residents would feel much more comfortable if medical rounds were scheduled regularly. One participant said that the regular scheduled medical rounds would prevent unnecessary hospital admissions.

*Participant 1:*” *...so it is of course useful for us to make these regular rounds and I also think that with all these hospital admissions, that ... at least how I see it ... “.*

*Participant 2: “It’s less, I think.” (Focus group 7).*

## Discussion

Results from the focus groups show that the implementation of CoCare is complex, and some modules were easier to implement than others. Participants perceived and described several barriers which had accounted for implementation difficulties. However, participants also reported facilitators and in some cases, how they overcame potential barriers. Furthermore, participants reported how barriers and facilitators may have influenced the outcome of CoCare.

Understaffing was seen as one of the biggest barriers for implementation and participants mentioned they could not comply with the intervention at all times. Previous reports had found that NH staff would have to work overtime to compensate for understaffing and bureaucracy [21]. Even though participants in this study did not mention overtime, the issue of understaffing and overtime is well-known in Germany with an average of 42 h of overtime per nurse within 6 months [22]. Furthermore, extended availability was not implemented in every NH due to understaffing, with some GPs refusing to do it due to a high workload. Again, this barrier could be overcome with more GPs being in charge of one NH.

Concerning bureaucracy, some participants feared the implementation of CoCare in standard care. For instance, bureaucracy was the reason why the CCC was not used properly, as it was perceived as complicated and double documentation was necessary to comply with regularly standards. Furthermore, the project CoCare itself accounted for a high administrative workload, with e.g. residents needed to give consent to receive the intervention. However, the dissolution of most of the bureaucracy can be expected once CoCare is implemented into standard care as the CCC would be recognized as the official documentation system and NH residents would no longer need to give consent.

In a scoping review, the intervention complexity was a common barrier for the implementation of integrated care [23], with rising complexity making it more difficult to implement the intervention [21]. In line with this, participants mentioned that CoCare and its implementation was complex and too detailed. For instance, some participants did not understand the differences between the case conferences, quarterly and annual meetings and therefore were not able to describe if these modules were conducted or implemented as part of CoCare in the NH. In addition, even though kick-off events and training were offered some participants did not know that the standard courses of treatment were available in a hard copy. Thus, a regular repetition of training, especially for new staff might be helpful to implement CoCare more thoroughly and to overcome potential barriers.

Yet, some barriers were not present within the project CoCare itself but were on a structural level. For instance, most NHs struggled to find medical specialists providing special medical care to residents. This problem is well known (e.g. Schäufele et al. [24]), with for example only 17% of NH residents having contact with an ophthalmologist within the time frame of a year in a previous study [25]. Legal options to improve medical care by specialists such as collaboration agreements between medical specialists and the association of statutory health insurance physicians *(Kassenärztliche Vereinigung)* seem to not have a sufficient effect [25].

All the barriers reported by the participants are well-known [23] and the implementation of complex health interventions remains a challenge [26]. Organizational and individual change processes are required for the implementation of health services interventions, particularly complex ones [21]. Completing these processes takes at least 1 year, or most likely longer [27]. With an implementation period of 33 months, CoCare provides a good time frame for full implementation and good incorporation into standard care. Furthermore, CoCare financially compensates for the higher workload for NHs and GPs and some barriers, such as the double documentation, will recede after implementation into regular care as described.

As expected, interprofessional communication and collaboration were perceived as one of the most important facilitators in CoCare according to the participants, with CoCare being seen as a team building intervention.

Pertinent German organizations recommend using tools, such as regularly scheduled medical rounds or extended availability to help improve interprofessional communication. Furthermore, nurses play a crucial role as they are the ones organizing interprofessional medical care in long-term care homes [28]. CoCare combines all these tools, providing a solid framework to facilitate interprofessional communication. Participants in our study valued the newly built teams of GPs and the contact persons within NHs. The implementation of a fixed contact person was not seen as useful in a previous qualitative study assessing interprofessional collaboration in nursing homes in Germany [29]. However, it was noted that the lack of perceived usefulness was due to NHs already having a fixed contact person for GPs before the intervention [29]. Thus, it can be hypothesized that a fixed contact person is perceived as useful when newly implemented in a NH.

Overall, interprofessional collaboration and communication were found to have an impact on health outcomes [30, 31]. For the elderly, interprofessional collaboration has positive effects e.g. on quality of life, mortality, or length of stay in hospitals [32]. Furthermore, there is some evidence that interprofessional collaboration is cost saving [33]. Some participants in this study had already concluded that the improved collaboration and communication prevented unnecessary hospital admissions and therefore had an impact on health outcomes for residents.

### Strengths and limitations

With *N* = 11 focus group interviews and a total of *N* = 74 participants, this qualitative study compromises a relatively big sample size.

Nonetheless, the study design displays some limitations. First of all, results were not distinguished between professionals, as the aim of this study was to analyze perceived barriers and facilitators by the CoCare team as a whole. However, doctors are known to evaluate collaboration and communication better than nurses within NHs [34–36] and future research should focus on that distinction.

Data saturation cannot be discussed, as no stop criteria was defined but a predetermined sample size was used. The predetermined sample size was based on the available NHs within the study. However, we would argue that with the abundance of material, data saturation was reached. As a further limitation, the different states of implementation within the participating NHs can be named. This might have affected the results, as some focus groups may not have talked about all barriers and facilitators. However, all barriers and facilitators described are well-known in the literature and thus, comparability to previous research is given.

## Conclusion

This study is only part of a more comprehensive process evaluation and thus a final conclusion of the implementation process cannot be drawn. Furthermore, the final evaluation of the intervention is still due. The quantitative, summative evaluation will be primarily based on claims data and questionnaires and will focus on the number of hospitalizations, as well as the residents` quality of life and health care costs.

Participants in this study describe several barriers for the implementation of a complex health intervention. However, some barriers would vanish if CoCare is implemented into routine care and also some barriers will be overcome by incorporation them into standard care overtime. Furthermore, participants describe communication and collaboration as the strongest facilitators for implementation and the project itself helped to improve this. Finally, participants perceived CoCare as potentially effective for the prevention of unnecessary hospitalizations of NH residents.

## Supplementary Information

**Additional file 1.**

## Data Availability

The datasets analyzed during the current study are not publicly available due to data protection regulations.

## Abbreviations

NH: Nursing Home
GP: General practitioner
CoCare: Coordinate Medical Care (Name of the project)
CCC: CoCare Cockpit (electronic medical record)
